# Non-coding RNAs mediate mechanical load-regulating bone metabolic homeostasis

**DOI:** 10.3389/fcell.2025.1643255

**Published:** 2025-10-23

**Authors:** Ruiqi Huang, Bo Chang, Xuejie Yi

**Affiliations:** ^1^ School of Physical Education, Liaoning Normal University, Dalian, Liaoning, China; ^2^ College of Exercise and Health, Shenyang Sport University, Shenyang, Liaoning, China; ^3^ College of Sports Science, Zhuhai College of Science and Technology, Zhuhai, Guangdong, China

**Keywords:** bone, mechanical load, non-coding RNA, micro RNA, bone disease

## Abstract

Bone, as a highly active organ, relies on dynamic mechanical stimulation for its continuous remodeling and regeneration. Mechanotransduction, the regulatory process through which mechanical forces are converted into biochemical signals, involves intricate interactions within and between cells and their extracellular environment, playing a crucial role in maintaining bone metabolic homeostasis. The complexity of this process stems primarily from the diversity of input signals and the precise regulation of downstream signaling cascades. In recent years, studies have revealed that non-coding RNAs (ncRNAs) play a key role in mediating the regulation of bone metabolism by mechanical loading. This review elaborates on how mechanosensitive ncRNAs participate in the regulation of bone mechanotransduction signaling pathways. Furthermore, we explore how different mechanical stimuli (such as loading and unloading) influence skeletal adaptive responses by modulating the expression of ncRNAs. Thus, this article not only provides novel perspectives on the mechanical regulatory functions of ncRNAs in bone metabolism, but also offers new strategies for preventing and treating bone metabolic disorders caused by mechanical disuse.

## 1 Introduction

Bone is a highly mineralized connective tissue that provides support while coping with enormous self-loads and responding to external mechanical stimuli. To meet these challenges, tight coupling between bone resorption by osteoclasts and bone formation by osteoblasts is essential for continuous bone remodeling events and maintaining bone homeostasis balance. Mechanical forces are essential for the ongoing bone-remodeling process.

Earlier, Wolff observed that bone growth, resorption, and remodeling are all related to the stress state and proposed the idea that skeletal adaptive responses occur in response to mechanical stress (Wolff, 1892; Wolff and Wolff, 1986). Frost later used the term “mechanical regulator” to describe and reveal how mechanical loading conserves bone mass and modulates bone adaptation mechanisms to prevent bone loss (Frost, 1987; Frost, 2000). Numerous studies have shown that mechanical stimulation, such as regular exercise, can promote bone anabolic responses and increase osteoblast proliferation and matrix precipitation to maintain bone homeostasis (Hsieh and Turner, 2001; Burr et al., 2002; Ozcivici et al., 2010; Sun W. et al., 2019). Conversely, in the absence of mechanical stimuli, such as long-term inactivity, disuse due to paralysis and bed rest, and long-term low-gravity exposure can reduce the ability of bone to perceive and withstand mechanical loads, leading to significant bone loss, which affect bone quality and increase the risk of bone-related diseases (Cauley and Giangregorio, 2020; Kliethermes et al., 2021; Donaldson et al., 1970; Bloomfield, 1997; Vico et al., 2000; Grimm et al., 2016; Vico and Hargens, 2018).

Common external mechanical loading stimuli include fluid shear stress, tensile stress and tension, compressive force and hydrostatic pressure, and microgravity associated with mechanical loading (Chen et al., 2010). Undoubtedly, these mechanical stimuli have been widely recognized as powerful factors driving cell physiological properties and determining the ultimate fate of cells. However, until now, the perception of the mechanical stimuli of the surrounding environment and the conversion of these mechanical force signals into biochemical signals to exert biological functions have always been a hot topic of research. Previous reports have revealed a variety of sensors that can sense and respond to bone mechanical signals, including primary cilia (Malone et al., 2007; Chen et al., 2016), focal adhesions (Haugh et al., 2015; Sato et al., 2020), connexins (Plotkin et al., 2015; Zhao et al., 2020), and ion channels (Lieben and Carmeliet, 2012; Ranade et al., 2015), which amplify signals from the outside into the cell through multiple pathways and directly activate downstream signals or alter cell morphology and behavior to regulate bone metabolism (Duncan and Turner, 1995; Moura et al., 2023).

Recently, increasing evidence has revealed the important role of non-coding RNAs (ncRNAs) in bone metabolism. Some ncRNAs also have mechanosensitive properties and are involved in mechanostimulus-induced bone metabolism. Understanding the underlying molecular mechanisms will help to better understand the functional roles of bone mechanics and bone biology. Therefore, in this review, we attempt to elucidate a series of biological processes and potential mechanisms by which ncRNAs mediate a variety of mechanical loads and participate in the regulation of bone metabolism to provide a new theoretical basis and potential therapeutic targets for the pathological mechanical environment to improve bone diseases.

## 2 Overview of ncRNAs

NcRNAs refer to RNA that does not have the potential to code for proteins. Based on their structure and function, they can be divided into structural and regulatory ncRNAs. Structural ncRNAs have well-established functions, while regulatory ncRNAs play a key role in regulating gene expression. Here, we focused on the functional role of regulatory ncRNAs, especially microRNAs, lncRNAs, and circRNAs, in mechanizing the regulation of bone metabolic homeostasis.

MicroRNAs (miRNAs) are approximately 22 nt endogenous RNAs that regulate gene expression by base-pairing with mRNA (Bartel, 2004). It is generally accepted that if the miRNA is perfectly complementary to the 3′non-coding region (3′UTR) of the mRNA, the former directs the mRNA to undergo a specific cleavage. However, when the two are not sufficiently complementary, miRNAs only guide translational inhibition and do not affect mRNA stability. Thus, once bound to the target gene, the miRNA acts post-transcriptionally through direct degradation of the target mRNA and/or inhibition of its protein expression (Guo et al., 2010; Bartel, 2018; Gebert and MacRae, 2019). In recent years, miRNAs have been recognized as important systemic regulators. It is estimated that miRNAs can regulate more than 30% of human protein-coding genes (Nilsen, 2007), and even regulate the environment in which genes are expressed within the cell. Importantly, it controls the expression of multiple target genes, resulting in a unified and unique biological response (Friedman et al., 2009). Long noncoding RNA (lncRNAs) are transcripts that are more than 200 nucleotides in length and are not translated into proteins. It is transcribed and processed like mRNA, and does not code for functional proteins (Nagano and Fraser, 2011; Ransohoff et al., 2018). Another abundant but mysterious class of RNAs is circular RNAs (circRNAs), which are formed by reverse splicing of mRNA or lncRNA exons. This type of molecule has a closed-loop structure, which is not affected by RNA exonucleases, and its expression is more stable and difficult to degrade. Although these ncRNAs operate by different mechanisms, according to the competitive endogenous RNA (ceRNA) mechanism, lncRNAs and circRNAs act as miRNA sponges, isolating miRNAs from targeted coding transcripts through shared miRNA binding sites, thereby preventing miRNAs from binding to target mRNAs and ultimately influencing a range of biological processes (Tay et al., 2014).

## 3 NcRNA regulate bone remodeling

Currently, the human genome appears to encode only 20,000–25,000 protein-coding genes (International Human Genome Sequencing Consortium, 2004). NcRNAs make up about 98% of the entire genome and account for about 60% of transcriptional output from human cells (Ambros, 2004; Yao Q. et al., 2024). This indicates their important role in physiological and pathological regulation. Accumulating evidence suggests that ncRNAs have emerged as important regulators of bone remodeling for bone growth and development, and the delicate coordination between osteogenic differentiation and osteoclast-mediated bone resorption (Lian et al., 2012; Ju et al., 2019; Aurilia et al., 2021; Huang et al., 2019; Puppo et al., 2021). In particular, the importance of miRNAs in regulating osteoblast-specific marker genes and controlling osteoblast differentiation *in vitro* has been widely demonstrated (Inose et al., 2009; Kapinas and Delany, 2011; Taipaleenmäki et al., 2012; Taipaleenmäki, 2018). With the continuous development of high-throughput analysis technology, there is an increasing number of reports on lncRNAs and circRNAs. Therefore, efforts are needed to reveal the key mechanisms of ncRNAs in the regulation of bone homeostasis.

In addition, researchers generally recognize the importance of the mechanistic environment in regulating bone structure and in determining the effectiveness of metabolic homeostasis (Ma et al., 2022; Ma et al., 2023). Although mechanotransduction is a process by which physical stimuli are transformed into biochemical reactions, the molecular mechanisms involved and their full scope remain unclear. It has been reported that changes in the microenvironment lead to activation of the mechanotransduction signaling pathway and its activity in response to the action of the underlying mechanosensor sensing the force (Wang et al., 2022; Qin et al., 2020). Although some important mechanotransduction pathways have been described in previous reports (Ozcivici et al., 2010; Liu et al., 2023), there is no doubt that ncRNAs will join these ranks (Yuan et al., 2017; Wang et al., 2018a; Cen et al., 2021). Unlike other traditional signaling hubs, multiple ncRNAs may simultaneously mediate one or more mechanical stimuli to accomplish the same instructions. However, ncRNA-mediated mechanical loading will further enhance the understanding of the mechanical forces on bone adaptation and provide new strategies for the diagnosis and treatment of metabolic bone diseases in the future.

## 4 Non-coding RNA mediates mechanical loading and regulates bone metabolism

### 4.1 *In vitro*: fluid shear stress

Bone tissue contains three pore sizes: the larger Volkmann and Haversian duct systems (∼20–50 μm), which contain blood vessels and interstitial fluid. The lacunar-canalicular system (LCS) (∼10 μm/∼0.1 μm) formed by the void outside the osteocyte with the tubule outside the osteocyte dendrite, which is a key determinant of bone adaptation in response to mechanical stimulation (van Tol et al., 2020), with a total surface area of approximately 215 m^2^ (Buenzli and Sims, 2015), and a tiny space (∼0.01 μm) between the smaller mineral hydroxyapatite and collagen fibers (Cowin and Cardoso, 2015). The mechanical load is an important regulator of bone structure and quality. In bone, macroscopic mechanical forces can cause tissue deformation; although it is difficult to visually observe and dissolve, the mineralized matrix generates an uneven pressure gradient under mechanical loading and drives interstitial fluids to flow through the lacunar tubular network and the porous mesh of the Haversian system (Piekarski and Munro, 1977; Fritton and Weinbaum, 2009), and load-induced fluids can generate greater flow velocities in the bone tissue to enhance migration, and the flow of these fluids creates shear stresses on the narrow pore tubing walls, thus affecting the biological microenvironment surrounding the cell (Riddle and Donahue, 2009). Thus, fluid shear stress (FSS) is the most common form of mechanical stimulation in the bone and is a major force in bone adaptation. Mechanosensitive cells sense the mechanical signals produced by FSS in the bone, thereby activating many signaling pathways and promoting cell proliferation and differentiation.

#### 4.1.1 FSS regulates osteocytes through ncRNA

Osteocytes, which account for 95% of the total bone cells, are embedded in the deep layers of the bone matrix and are an important component of mechanotransduction mechanisms (Qin et al., 2020). Mechanotransduction is a complex, multifaceted process that regulates multiple signaling cascades. In recent years, an increasing number of ncRNAs have been shown to be involved in the fluid shear stress in osteocytes. FSS (10 dyn/cm^2^) for one or 2 hours of OCY454 osteocyte-like cells activates the TGF-β signaling pathway (Dole et al., 2021). At the same time, FSS significantly inhibited miR-100 expression among the 61 mechanosensitive miRNAs identified (Dole et al., 2021). Consistent with previous reports (Zeng et al., 2012), this inhibition was sufficient to stimulate a multifold increase in osteogenic markers. The luciferase reporter gene showed that the Wnt signal transduction genes Fzd5 and Fzd8 are downstream targets of miR-100, but not Sost, a regulator that inhibits osteogenesis (Dole et al., 2021). These data suggest that miR-100 antagonizes Wnt signaling in osteocytes by reducing Wnt receptor expression. It behaves similarly to hematopoietic stem cells and breast cancer cells (Emmrich et al., 2014; Jiang et al., 2016). Thus, miR-100 mediates osteogenic differentiation mediated by fluid shear stress by tandem TGF-β and Wnt pathways.

The TGF-β and Wnt pathways are critically involved in regulating skeletal development and metabolic homeostasis. Although these pathways have been traditionally viewed as functionally independent, emerging evidence suggests a potential crosstalk between them. In human bone marrow stromal cells (hMSCs), TGF-β upregulates the expression of Wnt co-receptors and increases the accumulation and stability of β-catenin in the nucleus (Zhou et al., 2004). In addition, TGF-β also enhances Wnt signaling by regulating the modulation of axis inhibition protein 2 (Axin-2), which acts as a key regulator of the Wnt classical pathway (Gillespie et al., 2018). With the confirmation that miR-100 responds to FSS in osteocytes and mediates crosstalk between the two signaling pathways, the feedback loop of multi-signal synergy and the metabolic response mechanism of bone to mechanical load are further integrated. Notably, in this study, the authors emphasized the importance of the timing of total RNA collection after FSS (Dole et al., 2021).

As found by Govey et al., for osteocytes exposed to FSS *in vitro* (MLO-Y4), the maximum changes in transcription levels occurred at 2 h post-flow, while protein levels were maximally regulated after 8 h (Govey et al., 2014). In addition, the inhibition effect of samples collected 3 h after FSS on osteogenesis and osteoclastogenesis was more significant than that after 0 and 6 h (Yan et al., 2018). These findings highlight the temporal importance of mechanical stress transduction of osteocytes in regulating skeletal adaptive responses, especially because the cascade of signaling events triggered by fluid shear stress represents a cumulative effect. Of course, the MLO-Y4 cell line is not the same as primary osteocytes because of differences in the expression of certain genes and proteins. For example, the negative regulator of bone formation, scleroprotein Sost, does not appear to be expressed in MLO-Y4 cells (Yang et al., 2009).

#### 4.1.2 FSS regulates osteoblasts through ncRNAs

Previous studies have shown that FSS (10 dyn/cm^2^) on MLO-Y4 cells improved cell morphology, induced a good cytoskeleton, increased molecular viability, and was time-dependent on the expression of bone cell metabolism-regulating molecules (Yan et al., 2018; Xu et al., 2012). Interestingly, Xu et al. (2012) reported that this phenomenon was not observed in the MC3T3-E1 cells. Although the authors did not provide time for the collection of cells, the degree of surface chemical regulation of these superficial osteoblasts attached to the bone surface is affected by the sensitivity and tolerance of fluid shear stress, and these changes may depend on the cell adhesion function and the chemical reaction of the bone matrix (Li et al., 2016; Li et al., 2013). In contrast, numerous reports have shown that MC3T3-E1 osteoblasts respond to FSS and promote anabolic reactions through multiple pathways (Norvell et al., 2004; Liu et al., 2008; Mai Z. et al., 2013). Loading of FSS at 12 dyn/cm^2^ in MC3T3-E1 not only promoted the formation and rearrangement of actin stress fibers after 1 h but also facilitated the osteogenic differentiation process. In addition, miRNAs, including miR-19b, −20a, −21, −34a, −34c, -140-5p, and −200b, were significantly downregulated (Mai ZH. et al., 2013), suggesting that they may activate FSS-induced pre-osteoblast differentiation. This is because the expression levels of osteogenic differentiation biomarker genes (including Runx2, ALP, and Sp7) were significantly increased 12 h after FSS, and ALP activity was enhanced, as well as ARS staining for ECM mineralization. For miR-20a, it is a positive regulator of FSS-induced osteogenic differentiation. As predicted by bioinformatics, luciferase reporters showed that BAMBI and SMAD6 were direct targets of miR-20a. The latter activates the BMP2 signaling pathway by directly inhibiting the target genes BAMBI and SMAD6 to promote osteoblast differentiation (Peng et al., 2022). Similarly, miR-33-5p was identified as a novel mechanosensitive miRNA. miR-33-5p partially positively regulates the differentiation of MC3T3-E1 in osteoblasts by inhibiting the protein expression of Hmga2 at the post-transcriptional level (Runx2, Osx expression, and ALP staining are significantly upregulated) by 1 h, 10 dyn/cm^2^ FSS (Wang et al., 2016).

miRNA not only promotes osteoblast differentiation in response to FSS, but also plays a role in osteoblast proliferation and apoptosis. The expression of miR-34a in osteoblasts was reported to decrease continuously at an FSS of 12 dyn/cm^2^, reaching a minimum at 60 min (Wang et al., 2021). This promotes osteoblast proliferation and inhibits apoptosis (Wang et al., 2021). Subsequently, researchers focused on miR-34a in FGFR1, a member of the FGF family. Consistent with miR-34a, FGFR1 has the ability to mediate osteoblast proliferation and apoptosis (Sabbieti et al., 2009; McKenzie et al., 2019; Tuzon et al., 2019). Although the mRNA and protein levels of FGFR1 are significantly upregulated under FSS loading, miR-34a mimics only inhibited the protein levels of FGFR1 (Wang et al., 2021). In addition, the authors predicted that lncRNA TUG1 (Liu SC. et al., 2019; Hao et al., 2020), which is involved in the regulation of osteoblast proliferation and differentiation, may be an important potential target. The level of lncRNA TUG1 increases in a time-dependent manner under FSS induction and targets the FGFR1 pathway to promote osteoblast proliferation and inhibit apoptosis by sponging miR-34a (Wang et al., 2021). In the pathological state, the expression of lncRNA NEAT1 was elevated in the bone tissue and MC3T3-E1 osteoblast line of OVX mice exposed to FSS, whereas knockdown of lncRNA NEAT1 inhibited autophagy *in vitro* and *in vivo* (Zhao et al., 2022). Further studies have shown that lncRNA NEAT1 positively regulates HK2 via an endogenous competitive effect on miR-466f-3p (Zhao et al., 2022).

In general, although the lacunar network and vascular system of bone tissue are always full of fluid shear stress under the action of non-external forces, the continuous FSS simulated *in vitro* has a multi-effect on the bone microenvironment. In the case of ncRNAs, the competitive inhibition of miRNA, lncRNA, or both mediates FSS-induced mechanical signal transduction and promotes osteocyte proliferation and differentiation. It is not difficult to conclude that a short period of FSS *in vitro* is sufficient to improve bone parameters. Although it has been noted that the response of osteoblasts to low fluid shear stress is time-dependent (Ban et al., 2011). However, a single round of short-term fluid shear stress can induce osteogenic differentiation of MC3T3-E1 cells through crosstalk between integrin β1 and BMP2 signaling (Mai Z. et al., 2013). This reflects the high adaptability of bones to mechanical loads and the possible mechanical sensitivity to stable and/or transient FSS. On the other hand, a stress magnitude of 10–12 dyn/cm^2^ is the most prevalent, an important parameter for osteoblast activation, which is in line with the physiological range (8–30 dyn/cm^2^) previously considered by Weinbaum et al. (Weinbaum et al., 1994) and is sufficient to provide significant anabolic stimulation to the bones. In addition, osteocytes buried deep in the bone are embedded in the surface osteoblasts after terminal differentiation. Therefore, it is unclear whether the conduction of forces like FSS also increases the mechanical stimulation of bones from shallow to deep or whether the dendrites of osteocytes increase in response to shear stress (Zhang et al., 2006) without cross-interference with bone surface cells. In the future, there is a need for in-depth studies of different intercellular crosstalk induced by mechanical forces in bone to characterize how the bone microenvironment is affected by mechanical loading, especially with regard to the pleiotropy of ncRNAs in bone. At the same time, the complexity of the microenvironment also indicates the therapeutic target potential of the mechanotransduction pathway, and the elucidation of the ncRNA and protein encoded by the target gene that are differentially expressed in response to fluid flow is conducive to the clinical design of reasonable drugs for bone diseases (Table 1).

**TABLE 1:** Fluid shear stress regulates the effects of ncRNAs on bone tissue or cells.

Species	Cell type	Mechanical loading conditions	ncRNAs	Target gene	Phenotype
Mouse	OCY454 (Dole et al., 2021)	10 dyn/cm^2^	miR-100 ↓	FZD5 FZD8	osteogenic differentiation↑
Mouse	MC3T3-E1 (Mai et al., 2013)	1h, 12 dyn/cm^2^	miR-19b, −20a, −21, −34a, −34c, -140-5p, −200b ↓	n.s	osteogenic differentiation↑
Mouse	MC3T3-E1 (Peng et al., 2022)	1h, 12 dyn/cm^2^	miR-20a ↓	BAMBI SMAD6	osteogenic differentiation↑
Mouse	MC3T3-E1 (Wang et al., 2016)	1h, 10 dyn/cm^2^	miR-33-5p ↑	Hmga2	osteogenic differentiation↑
Mouse	MC3T3-E1 (Wang et al., 2021)	1h, 12 dyn/cm^2^	lncRNA TUG1 ↑ miR-34a ↓	FGFR1	osteoblast proliferation↑ apoptosis↓
Mouse	MC3T3-E1 (Zhao et al., 2022)	45min, 12 dyn/cm^2^	lncRNA NEAT1 ↑ miR-466f-3p ↓	HK2	autophagy↑

FZD5, frizzled class receptor 5; FZD8, frizzled class receptor 8; BAMBI, BMP, and activin membrane-bound inhibitor; SMAD6, SMAD, family member 6; Hmga2, High mobility group protein A2; FGFR1, fibroblast growth factor receptor 1; HK2, hexokinase 2.

### 4.2 *In vitro*: tensile stress/tension

According to previous theories, mechanical forces exerted on bones generate two local mechanical signals on the cell: deformation of the extracellular matrix and extracellular fluid flow (Owan et al., 1997). Unlike fluid shear forces, mechanical tensile stress tends towards the former, which is an internal force between different parts of the object that can resist external factors and the effects that cause the deformation of the object to a certain extent. Mechanical tensile stress is essential for various tissues that are constantly subjected to mechanical loads and function properly.

#### 4.2.1 Tensile stress regulates osteocytes and osteoblasts through ncRNA

In the bone, osteocytes act as highly sensitive mechanosensors and regulators, integrating mechanosensory stimuli to form biochemical signals, modulating skeletal representations through their own dendritic structure, and secretion of biochemical factors from pore tubules regulate skeletal remodeling (Qin et al., 2020; Delgado-Calle and Bellido, 2022). Osteoblasts, on the other hand, are located on the surface of the bone and are defined as cells that form the bone matrix, and their structure and function adapt rapidly to external forces. Although both are key cells involved in sensing and communicating changes in bone structure or mass owing to changes in loading, their adaptations to mechanical loading responses differ. In osteoblast cells, Guo et al. (Guo et al., 2015) stimulated MC3T3-E1 cells to 0.5 Hz and 2,500 με for multiple hours under mechanical tensile strain, and the 8-h strain was most beneficial for promoting osteogenic differentiation compared to other times. Four mechanically reactive miRNAs were identified simultaneously. Subsequently, the team applied MLO-Y4 osteocytes to mechanical tensile strain under the same conditions (Zeng et al., 2019). They identified miR-29b-3p, which had the most significant downregulation after stretching. Previous studies have shown that overexpression of miR-29b-3p in MC3T3-E1 osteoblasts promotes osteogenic differentiation (Li et al., 2009). However, miR-29b-3p did not respond to stretched osteoblasts. In contrast, in osteocytes, stretching inhibits the expression of miR-29b-3p and negatively regulates its target gene, insulin-like growth factor IGF-1 (Zeng et al., 2019). Notably, osteocytes exposed to mechanical strain in their conditioned medium promoted the osteogenic differentiation of MC3T3-E1 cells. On the other hand, overexpression of miR-29b-3p inhibits osteoblast differentiation by decreasing IGF-1 levels in MLO-Y4 osteocyte-conditioned medium used to culture MC3T3-E1 cells (Zeng et al., 2019). These results suggest that the crosstalk between the two may regulate IGF-1 secretion in osteocytes through mechanosensitive miRNAs, thereby guiding osteoblast differentiation. Adipose-derived mesenchymal stem cell ADSCs were extracted from 6-week-old mice under similar conditions (0.5 Hz, 2000 με, 2 h/day), and the 3′UTR of Wnt11 in the non-canonical Wnt pathway was found to have seed-matched sites with the screened miR-154-5p after cyclic uniaxial tensile strains. The expression of miR-154-5p was downregulated in a time-dependent manner in tensile-stressed cells. Conversely, overexpression of miR-154-5p inhibits Wnt11 at the post-transcriptional level (Li et al., 2015). These results suggest that miR-154-5p negatively regulates the osteogenic differentiation of ADSCs and mediates it by activating the non-canonical Wnt pathway RhoA-ROCK (Li et al., 2015). Due to its important role in regulating cytoskeletal dynamics, this pathway enables the osteogenic fate of stem cells by triggering intrinsic cellular tension (McBeath et al., 2004; Chen and Jacobs, 2013; Saidova and Vorobjev, 2020).

In a study using the human osteoblast line hFOB1.19, Zuo et al. (2015) found that three miRNAs were expressed and seven miRNAs were reduced after 3 days of cyclic mechanical stretching (at 0.5 Hz frequency) (Zuo et al., 2015). Among them, the significantly downregulated miR-103a mimic had the most significant inhibitory effect on luciferase reporter activity and bound to the 3′UTR of Runx2 mRNA to reduce its protein level and prevent osteogenic differentiation. In contrast, mechanical stretching enhances the expression of osteoblast marker genes as well as the deposition of calcareous nodules. This may be due to a significant rearrangement of the cytoskeletal orientation due to stretching (F-actin) but has no effect on cell proliferation (Zuo et al., 2015). Interestingly, mechanical stretching not only activates the ERK1/2 and Wnt/β-catenin signaling pathways but also upregulates Runx2 protein, but not mRNA expression. Similarly, the miR-103a mimic significantly reduced the protein expression of Runx2 (Zuo et al., 2015). Under cyclic stretch stimulation, upregulation and downregulation of miR-103a expression suppressed and promoted osteogenic differentiation, respectively. However, when miR-103a mimics and inhibitors were co-transfected with siRunx2, the function of the miR-103a oligonucleotide was completely blocked, whereas the osteogenic marker gene remained at a low level, suggesting that the function of miR-103a in cyclic mechanical stretching-induced osteoblast differentiation was Runx2-dependent (Zuo et al., 2015).

It is important to emphasize that in order to realistically simulate the physiological level *in vivo*, the authors first determined by finite element analysis that the strain loaded at the level of the proximal femur bone was approximately 815 ± 57 με (Zuo et al., 2015). It is a powerful tool for studying bone structure and assessing mechanical properties (Meslier and Shefelbine, 2023). Based on the loading amplification mechanism identified by Cowin et al. (Cowin and Weinbaum, 1998) and You et al. (2001), that is, when the mechanical load is transferred from the tissue level to the cellular level, the whole tissue strain needs to be amplified 10–100 times to elicit an *in vitro* biochemical reaction. Therefore, they ultimately chose to use a microstress of 80,000 με for simulation intervention (Zuo et al., 2015). Exercise-induced tissue-level strain has been reported to be no more than 0.2% (2000 με) and may cause damage to bone tissue *in vivo* when deformation exceeds 0.5% (Burr et al., 1996; Fritton et al., 2000). *In vitro* studies have shown that more than 0.5% tissue-level strain is required to initiate intracellular signaling (You et al., 2000). This reflects the large differences in the adaptive regulation of mechanical external forces by units at different levels in the body. However, the 80,000 με value is far from the previously described 2000/2,500 με (Guo et al., 2015; Zeng et al., 2019; Li et al., 2015). Yan et al. (2012) exposed mouse MC3T3-E1 pre-osteoblasts to mechanical tensile strain and examined the proliferative activity of cells under different mechanical strains (1,000, 1,500, 2000, and 2,500 με on a 0.5 Hz basis), with 2000 and 2,500 με also significantly promoting cell proliferation (Yan et al., 2012). In line with this, 2,500 με promotes osteoblast differentiation and inhibits osteoclast activity (Liu et al., 2012). Mechanical stress of 5,000 με enhanced the percentage of PI-positive staining and lactate dehydrogenase (LDH) activity in the cell culture medium, suggesting that cells were overloaded and apoptotic under this strain, which is clearly a mechanical environment that cells cannot withstand (Yan et al., 2012). Similarly, Frost believed that bone strain in the peak range of 1,500–3,000 με would promote bone remodeling and increase the bone mass. A value of 5,000 με is the critical point for physiological and pathological strain in bone (Frost, 1987). Although there are still different voices for this parameter. However, the bones may withstand 10,000–30,000 με. This means that the bones may be in overload condition on a regular basis. Overloading may lead to pathological bone remodeling, microinjury, and even fractures.

#### 4.2.2 Tensile stress regulates mesenchymal stem cells via ncRNA

Mesenchymal stem cells (MSCs) are mechanostimulus-sensitive cells with multiple differentiation potential. The differentiation process requires complex network signal conditioning, which can be effectively controlled by mechanical loading and ncRNAs (Gangaraju and Lin, 2009; Sarraf et al., 2011; Hao et al., 2015). In terms of stress, in addition to the tensile stress of the object expressed by the strain (ε) or microstrain (με), the deformation rate, that is elongation, can also be used as another method of expression. As described by Chung and Rylander, (2012) and Flxecell’s website, tensile elongations of 1% and 10% correspond to 0.01 and 0.1 strains (ε) of respectively. The Flexcell tensile stress system is widely used in vitro mechanistic models to explore the level of superphysiological stress on cells.

Performing 6 h of daily mechanical stretching (10%, 0.5 Hz) in hBMSC resulted in mechanosensitive lncRNA H19 and miR-138, respectively, and mediated osteogenic differentiation of hBMSCs. Further studies have shown that lncRNA H19 forms ceRNAs with miR-138 and targets PTK2 (Wu et al., 2018). The latter gene encoding focal adhesion kinase (FAK). Knockdown of lncRNA H19 relaxes its endogenous competition for miR-138 and inhibits FAK expression; however, this inhibition is rescued by downregulating miR-138 under mechanical stretch conditions (Wu et al., 2018). Consistently, mechanical strain of primary extracted BMSCs for 7 days at 4 h per day, 6% deformation intensity, and 0.5 Hz showed that the expression of lncRNA H19 was upregulated after tensile strain, while the Wnt/β‐catenin pathway obstruction, ALP activity, and osteogenic differentiation inhibition caused by knockdown of lncRNA H19 were alleviated by mechanical strain (Liu et al., 2024). The involvement of lncRNA H19 in osteogenic differentiation has been reported (Zhou Z. et al., 2021). For miR-138, the expression of miR-138 in bone tissue was significantly increased 4 weeks after hind limb unloading in 8-week-old mice (Chen et al., 2020). These results indicated that miR-138-5p is a mechanoreactive miRNA that is negatively correlated with bone formation. This finding is consistent with the conclusions of previous predecessors (Eskildsen et al., 2011). Primary osteoblasts from wild-type mice were subjected to a cyclic mechanical stretch of 1 Hz for 12 or 24 h at 10% deformation *in vitro*. It was found that miR-138-5p levels continued to decrease, whereas osteoblast differentiation capacity was enhanced (Chen et al., 2020). Compared to WT osteoblasts, the increase in osteoblast-specific miR-138-5p transgenic (TG) mouse primary cells constructed by prokaryotic microinjection was limited. In contrast, the ability of osteoblasts to differentiate was significantly restored after treatment with miR-138-5p antagonist and exposure to mechanical stress conditions (Chen et al., 2020).

BMSCs isolated from the femur and tibia of Wistar rats were screened for mechanosensitive miR-503-5p using miRNA microarray assay under stretch induction (10%, 1 Hz, 12 h). It is inversely correlated with the osteogenic marker genes, Runx2 and ALP (Liu et al., 2017). Overexpression of miR-503-5p in BMSCs attenuates stretch-induced osteogenic differentiation, while inhibition of miR-503-5p reverses this effect (Liu et al., 2017). Similarly, BMSCs isolated from the bone tissue of SD rats promoted tensile strain (5%, 0.5 Hz, and 6 h/day)-induced osteogenic differentiation, and inhibited adipogenic differentiation after overexpression. Silencing lncRNA MEG3 showed the opposite trend. Dual-luciferase reporter results support the lncRNA MEG3-targeted inhibition of miR-140-5p (Zhu et al., 2021). Likewise, previous reports have shown that lncRNA MEG3 inhibits adipogenesis and promotes osteogenesis of human adipose-derived mesenchymal stem cells (hADSCs) via miR-140-5p (Li Z. et al., 2017). However, in BMSCs stimulated by tensile strain, overexpression of lncRNA MEG3 resulted in a significant decrease in miR-140-5p expression and promotion of osteogenic differentiation, whereas silencing lncRNA MEG3 induced the opposite effect (Zhu et al., 2021).

Another study on the regulation of bone marrow mesenchymal stem cell differentiation by circulating tensile strain showed that tensile strain was induced at 10% elongation for 7 days for 2 h per day at 1 Hz. The expression of 40 miRNAs was altered after the cyclic stretching treatment. Among these, miR-365 was the most upregulated miRNA. Inhibition of the expression of this miRNA inhibits the expression of marker genes and proteins (cartilage-associated factors Col2a1, ANCN, and SOX9) at chondrogenic levels, and this effect was abrogated by cyclic mechanical stretching induction (Chen and Wu, 2019). Luciferase has reported that miR-365 targets histone deacetylase HDAC4, a known negative regulator of chondrocyte differentiation that controls chondrocyte hypertrophy during bone production (Vega et al., 2004). The expression of HDAC4 was verified using miR-365 simulant. In conclusion, cyclic mechanical stretching-activated miR-365 promotes chondrogenic formation of BMSCs by inhibiting its target genes (Chen and Wu, 2019). Similarly, several other miRNAs have been shown to regulate BMSC chondrogenesis (Ham et al., 2012; Lin et al., 2014); however, the role of these miRNAs under mechanical stress remains unknown.

#### 4.2.3 Tensile stress regulates bone metabolism through ncRNA in pathological conditions

In addition to promoting chondrogenesis, miR-365 overexpression can also target HDAC4 to inhibit endplate chondrocyte degeneration (Zheng et al., 2019), a major factor in intervertebral disc degeneration. Studies have shown that appropriate short-term stimulation can increase the synthesis of the cartilage extracellular matrix (Xu et al., 2016), whereas excessive loading can degrade the extracellular matrix and induce cartilage endplate degeneration (Zhu et al., 2019). Xiao et al. (2020) showed that circRNA 0058097 promotes tension-induced cartilage degeneration by sponging miR-365a-5p. They also observed downregulation of circRNA 0022382 (circ FADS2) at the same tension (0.5 Hz, 10%, and 8 h). However, overexpression of circ FADS2 upregulates the expression of its target gene TGF-β3 by competing for miR-4726-5p to improve intervertebral disc degeneration induced by excessive tension (Hu et al., 2022). Similarly, a previous study reported that 10% stretching induction of endplate chondrocytes at a cyclic tension of 0.5 Hz (8 h per day) promoted cell proliferation but did not affect cell apoptosis. The arrangement of the F-actin cytoskeleton in the cell changes significantly after loading, and the cell gradually changes from polygonal to long spindle shape (Xiao et al., 2018). 23 differentially expressed miRNAs were screened and validated for three miRNAs that were also under-expressed in degenerative diseases: miR-125a-5p, miR-455-5p, and miR-199a-5p. miR-455-5p has a Runx2 binding site (Xiao et al., 2018). Overexpression or inhibition of miR-455-5p alters the expressions of Runx2, MMP13, COLX, COL2, and SOX9 under the action of force (Xiao et al., 2018). This suggests that miR-455-5p can alleviate stretch-induced endplate chondrocyte degeneration by targeting Runx2.

As with tension in the IDD, mechanical tensile stress is not entirely beneficial for osteogenic differentiation. When MC3T3-E1 osteoblasts were cultured at 0.1 Hz, 12% cyclic tensile stress for 24 h, miR-132-3p expression was upregulated approximately 6-fold in MC3T3-E1 cells, while ALP activity and Ocn expression were decreased (Liu M. et al., 2019). miR-132-3p was the only significant miRNA associated with Smads signaling among the 44 differentially expressed genes validated by RT-qPCR. Although this tensile stress reduces the phosphorylation levels of Smad2 and Smad5 in MC3T3-E1 cells. However, only protein expression of Smad5 is observed, and not mRNA inhibition after overexpression of miR-132-3p is observed. Luciferase reporter assay demonstrates that Smad5 is a direct target of miR-132-3p (Liu M. et al., 2019). These results suggest that miR-132-3p inhibits osteoblast differentiation by downregulating the translation of Smad5 in mouse osteoblasts under mechanical stretch induction. However, based on previous descriptions, Hu et al.(2015) suggested that miR-132-3p could be a promising new therapeutic target to prevent microgravity-induced reduction in bone formation. Although these reports suggest that miR-132-3p negatively regulates osteogenesis, the upregulation of miR-132-3p in hindlimb unloaded bone tissue (Hu et al., 2015; Hu et al., 2020), is diametrically opposed to the change *in vitro* mechanical stress (Liu M. et al., 2019). This suggests that there may be differences in the expression of the same miRNA under different forms of mechanical loading. In addition, tensile strain reduces the expression of circ Strn3 in chondrocytes and stimulates chondrocytes to secrete exosomal miR-9-5p, which inhibits osteoblast differentiation through endogenous competition and targeting Kruppel-like factor 5 (KLF5) (Li et al., 2023). *In vivo*, intra-articular injection of exosomal miR-9-5p significantly alleviates OA progression in mice (Li et al., 2023).

These studies on cell deformation have shown that although there are some differences in mechanical stretching parameters, in general, these bone-related cells have withstood the challenge of mechanical stimulation *in vitro* and met their physiological needs to achieve bone health under the action of ncRNA. Although there are currently multiple instruments that help us to simulate the mechanistic environment *in vitro* and better understand mechanistic signaling and uncover the underlying molecular mechanisms. However, from a practical point of view, in addition to the need to solve the pathological bone remodeling disorder and clinical manifestations, considering many factors such as the position, direction and depth of the force, there is still a need for future efforts to elucidate the magnitude and duration of mechanical loads in different cellular environments *in vitro*. In addition, the difference between *in vivo* and *in vivo* strains must be considered in order to more accurately identify *in vivo* forces. After all, the physiological environment *in vivo* is extremely complex, and different types of cells have different activities, response rates, and localization and expression in tissues. In general, osteocytes, osteoblasts, and mesenchymal stem cells are gradually being revealed to sense mechanical cues and respond adaptively to regulate bone metabolic homeostasis through ncRNAs (Table 2).

**TABLE 2:** Stretching stress regulates the effects of ncRNAs on bone tissue or cells.

Species	Cell type	Mechanical loading conditions	ncRNAs	Target gene	Phenotype
Mouse	MC3T3-E1 (Guo et al., 2015)	0.5 Hz, 2500 με, 8 h	miR-218, -33↓ miR-191, -3070a↑	n.s	osteogenic differentiation↑
Mouse	MLO-Y4 (Zeng et al., 2019)	0.5 Hz, 2500 με, 8 h	miR-29b-3p ↓	IGF-1	osteogenic differentiation↑
Mouse	ADSC (Li et al., 2015)	0.5 Hz, 2000 με, 2 h/d	miR-154-5p ↓	WNT11	osteogenic differentiation↑
Human	hFOB1.19 (Zuo et al., 2015)	0.5 Hz, 8%	miR-103a ↓	RUNX2	osteogenic differentiation↑
Human	hBMSC (Wu et al., 2018)	0.5 Hz, 10%, 6 h	lncRNA H19 ↑ miR-138 ↓	PTK2	osteogenic differentiation↑
Mouse	BMSCs (Liu et al., 2024)	0.5 Hz, 6%, 4h, 7d	lncRNA H19 ↑	n.s	osteogenic differentiation↑
Mouse	OBs (Chen et al., 2020)	1 Hz, 10%, 12/24 h	miR-138-5p ↓	MACF1	osteogenic differentiation↑
Rat	BMSCs (Liu et al., 2017)	1 Hz, 10%, 12 h	miR-503-5p↓	n.s	osteogenic differentiation↑
Rat	BMSCs (Zhu et al., 2021)	0.5 Hz, 5%, 6 h/d	lncRNA MEG3 ↑ miR-140-5p ↓	n.s	osteogenic differentiation↑ adipogenic differentiation↓
Rat	BMSCs (Chen and Wu, 2019)	1 Hz, 10%, 2h, 7d	miR-365 ↑	HDAC4	chondrogenic differentiation↑
Human	Endplate chondrocytes (Xiao et al., 2020)	0.5 Hz, 10%, 8 h	circRNA 0058097↑ miR-365a-5p↓	HDAC4	endplate cartilage↑
Human	Endplate chondrocytes (Hu et al., 2022)	0.5 Hz, 10%, 8 h	circRNA FADS2↓ miR-4726-5p↑	TGF-β3	endplate cartilage↑
Human	Endplate chondrocytes (Xiao et al., 2018)	0.5 Hz, 10%, 8 h	miR-455-5p↓	RUNX2	endplate cartilage↓
Mouse	MC3T3-E1 (Liu et al., 2019b)	0.1 Hz, 12%, 24 h	miR-132-3p ↑	SMAD5	osteogenic differentiation↓
Human	Chondrocytes (Li et al., 2023)	5 Hz, 20%, 12–48 h	circRNA Strn3 ↓ miR-9-5p ↑	KLF5	osteogenic differentiation↓

IGF-1, insulin-like growth factor-1; WNT11, Wnt family member 11; RUNX2, runt related transcription factor 2; PTK2, protein tyrosine kinase 2; MACF1, microtubule actin crosslinking factor 1; HDAC4, histone deacetylase 4; TGF-β3, transforming growth factor beta 3; SMAD5, SMAD, family member 5; KLF5, Kruppel-like factor 5.

### 4.3 *In vitro*: compressive force and hydrostatic pressure

In contrast to the tension of tension, compressive force is the compression of an object, but it seems difficult to observe in rigid bones. Needless to say, one of the most important functions of bones is to provide support. Weight-bearing bones are constantly under the pressure of their own weight, which bones seem to have long been accustomed to. Perhaps because of this, more attention has been paid to the response of bones when they lose weight or are exposed to other mechanical stimuli, and the effects of compressive force on bones have been neglected. Studies have shown that compression is a rate-limiting process, and excessive pressure is detrimental to bone tissue. However, the controversial results of stress deserve in-depth exploration. Previous studies have shown that a pressure of 1.0 g/cm^2^ is the optimal condition for MC3T3-E1 osteogenic differentiation (Yanagisawa et al., 2008), and that osteogenic differentiation increases with increasing pressure (Tripuwabhrut et al., 2013), until the upper limit of the effect of pressure on cell viability (5.0 g/cm^2^) (Shen et al., 2017). Conversely, applying a compressive force of 3.0 g/cm^2^ (294 Pa) for 24 h slowed the growth of osteoblast MC3T3-E1 compared to the non-stressed group (Iwawaki et al., 2015). RT-qPCR after microarray analysis showed that compressive force increased the level of miR-494-3p, while inhibition of cell proliferation was attributed to a decrease in miR-494-3p′s direct target fibroblast growth factor receptor 2 (FGFR2) and Rho-associated coiled coil kinase 1 (ROCK1) (Iwawaki et al., 2015). However, osteogenic differentiation of MC3T3-E1 was inhibited by downregulating the expression of miR-494-3p in senescent MLO-Y4 osteocyte-derived exosomes (Yao C. et al., 2024). Although miR-494-3p targeted a number of other genes this time, these results show that regardless of miR-494-3p expression, it appears to be detrimental to osteogenesis. However, the expression of lncRNA PAGBC was increased by 100 psi hydrostatic pressure, and miR-133b was inhibited in the form of competitive endogenous RNA, thereby promoting osteogenic differentiation of adipose mesenchymal stem cells (AMSCs) by upregulating the expression of Runx2 (Ru et al., 2020).

Due to the high water content of cartilage tissue (about 70%), the main loading stimulus sensed by chondrocytes embedded in the cartilage matrix is hydrostatic pressure (Sun, 2010; Liu S. et al., 2019), and the physiological range in the body is usually 0–10 MPa (Chen et al., 2013). It has been shown that the physiological circulatory load on articular cartilage is fundamental to the regulation of chondrocyte metabolic activity (Segarra-Queralt et al., 2024). Articular cartilage degeneration and periarticular and subchondral bone thickening are clinical features of osteoarthritis OA (Martel-Pelletier et al., 2016). To simulate the most common knee joint pressure (5 MPa) during normal gait (Guilak and Hung, 2005), Cheleschi et al. exposed chondrocytes from normal individuals and OA patients to circulating hydrostatic pressure (1–5 MPa, 0.25 Hz) for 3 h and collected cells at different time points within 48 h (Cheleschi et al., 2017). It was found that the expression levels of miR-27a/b, miR-140, and miR-146a were significantly increased in OA chondrocytes, and the Wnt/β-catenin pathway was activated by inhibiting their target genes (Cheleschi et al., 2017). Moderate HP induction plays a positive role in OA cells by restoring certain miRNA levels. Subsequently, the team also observed a significant increase in miR-155 and miR-181a in OA cells, but both were immediately downregulated under the same pressure protocol (De Palma et al., 2018), while high continuous HP (24 MPa) reversed the expression of miR-155 and miR-181a (Cheleschi et al., 2021). Previously, it was found that excessive pressure (25 MPa) induced changes in gene expression in mouse ATDC5 chondrocyte progenitor cells were similar to those observed in OA cartilage (Montagne et al., 2017). Excessive mechanical pressure can damage the extracellular matrix of chondrocytes and alter the balance of chondrocytes. Subsequently, in order to better characterize the effect of miR-155 on cartilage homeostasis, Montagne et al. (2022) subjected ATDC5 cells to 5 MPa, 10 MPa, and 25 MPa pressure. Unlike previous studies, they found that Mir155 hg was significantly upregulated only at high HP (25 MPa) after applying pressure for up to 48 h (Montagne et al., 2022). Mir155 hg is the host gene of miR-155 and belongs to a type of lncRNA. Importantly, Mir155 hg begins to upregulate as early as 1 h after pressurization and has since maintained a high level. However, upregulation of low abundance chain miR-155-3p and high abundance chain miR-155-5p processed from Mir155 hg is not significantly induced until at least 24 h later (Montagne et al., 2022). This may be attributed to the regulation and subcellular localization of lncRNA, as Mir155 hg is mainly localized within the nucleus (Montagne et al., 2022). In addition, the upregulation response induced by high HP seems to activate some membrane channels, further confirming previous views that HP may alter membrane fluidity or increase membrane bending stiffness to alter membrane forces, thereby triggering mechanical transduction and indirectly affecting the conformation and binding of signaling molecules (Montagne et al., 2014; Purushothaman et al., 2015). In addition, the abnormal compressive force regulates several miRNAs in the articular cartilage (Stadnik et al., 2021). *In vitro*, when bovine full-depth articular cartilage explants were loaded to 2.5 or 7 MPa (1 Hz, 15 min), it was observed that miR-221, miR-222, miR-21-5p, and miR-27a-5p were all higher than 2.5 MPa at 7 MPa, while miR-483 levels were opposite (Stadnik et al., 2021). These miRNAs regulate cartilage homeostasis by recognizing the magnitude of the load and inhibiting downstream targets. Based on previous views (≤6 MPa is usually accepted as physiological load) (Fehrenbacher et al., 2003), they believe that the compression force of 7 MPa exceeds the physiological range. Although this range is inconsistent with the previous description, it has to be admitted that the mechanical regulation under this “non-physiological” (7 MPa) is significantly better than the physiological 2.5 MPa. Therefore, the scoping of mechanical forces under physiological and non-physiological conditions still needs to be standardized among different species. In short, the maintenance of articular cartilage homeostasis depends on chondrocytes, and also depends on the regulation of mechanical load, especially under dynamic compressive force, the balance of its anabolic and catabolic activities is maintained so that the cartilage extracellular matrix is in a constant state of renewal to adapt to the moderate mechanical load challenge, which has a certain preventive and slowing effect on the occurrence and development of osteoarthritis.

However, like excessive tension, high compressive force can also induce NP damage to nucleus pulposus cells in the progression of intervertebral disc degeneration (IDD) to exacerbate the condition. Intervertebral discs are extremely sensitive and fragile in response to pressure. The pressure range ranges from 0.1 MPa to 2.3 MPa, with a pressure of 0.5 MPa when standing relaxed (Wilke et al., 1999). Treatment of human NP cells under 1.0 MPa pressure stimulation for 36 h was sufficient to cause damage, and the expression of circ CIDN and circ CDR1as was significantly decreased. They bind to miR-34a-5p and miR-432-5p, respectively, and target SIRT1 and SOX9 to attenuate compression-load-induced apoptosis in NP cells (Xiang et al., 2020; Xiang et al., 2023). These results suggest that circRNA may provide a new approach for the prevention and treatment of endplate cartilage degeneration and IDD.

In summary, the supportive effect of mechanical stress on bones is a sophisticated and dynamic physiological process, whose core lies in the conversion of external mechanical forces into biochemical signals by bone cells, thereby driving bone synthesis metabolism and maintaining homeostasis. *In vitro* mechanical forces, including shear stress and cyclic tensile stress and pressure, can modulate the expression of a panel of ncRNAs that are involved in the cellular response to mechanical forces in different cell lines. For shear stress and tensile stress, current data show that osteocytes share a common effect on the mechanotransduction perceived by the exoskeleton, and that moderate stress loading is essential for osteogenic differentiation. Compressive force, on the other hand, is common in OA and IDD-associated chondrocytes and exhibits heterogeneity. In addition, the perception and adaptation of different ncRNAs in bone tissue to force may depend on the magnitude of the force and the location of the force. However, the effect of loading within the physiological range on the promotion of osteogenic differentiation and the improvement of bone diseases reflects the importance of mechanical load on bone. Although these simulations of *in vitro* mechanical stimulation do not realistically reproduce the scene under physiological conditions, they show at least at the microscopic level how the bone microenvironment can cope with the challenges of different forms of mechanical loading to meet the physiological demands of the skeleton. Together, the mechanical loads of these contacts play a crucial role in mechanistic signaling and subsequent regulation of bone remodeling and homeostasis (Table 3).

**TABLE 3:** Compressive stress/Hydrostatic pressure regulates the effects of ncRNAs on bone tissue or cells.

Species	Cell type	Mechanical loading conditions	ncRNAs	Target gene	Phenotype
Mouse	MC3T3-E1 (Iwawaki et al., 2015)	3.0 g/cm^2^, 24 h	miR-494-3p ↑	FGFR2 ROCK1	cell proliferation↓
Human	AMSC (Ru et al., 2020)	100psi	lncRNA PAGBC ↑ miR-133b ↓	RUNX2	osteogenic differentiation↑
Human	Chondrocytes (Cheleschi et al., 2017)	0.25Hz, 1–5MPa, 3 h	miR-27a/b −140, −146a ↑	MMP13 ADAMTS5 HDAC4	Wnt/β-catenin pathway↑
Human	Chondrocytes (De Palma et al., 2018)	0.25Hz, 1–5MPa, 3 h	miR-155, -181a↓	n.s	n.s
Human	Chondrocytes (Cheleschi et al., 2021)	24 MPa	miR-27a, −140↓ miR-34a, −146a −155, −181a, -let7e ↑	n.s	n.s
Mouse	ATDC5 (Montagne et al., 2017, Montagne et al., 2022)	25MPa, 1h/24 h	Mir155 hg ↑ miR-155-3p, 155-5p↑	n.s	cell growth↓ cartilage marker↓
Bovine	Bovine full-depth articular cartilage (Stadnik et al., 2021)	1Hz, 2.5/7 MPa 15min	miR-483↓ miR-221, -222 −21-5p, −27a-5p ↑	TIMP3 CPEB3	cartilage homeostasis
Human	Nucleus pulposus cell (Xiang et al., 2020)	1.0MPa, 36 h	circRNA CDR1as ↓ miR-432-5p ↑	SIRT1	NP cell damage
Human	Nucleus pulposus cell (Xiang et al., 2023)	1.0MPa, 36 h	circRNA CIDN↓ miR-34a-5p↑	SOX9	NP cell apoptosis

FGFR2, fibroblast growth factor receptor 2; ROCK1, rho-associated coiled-coil kinase 1; RUNX2, runt related transcription factor 2; MMP13, matrix metallopeptidase 13; ADAMTS-5, ADAM, metallopeptidase with thrombospondin type 1 motif 5; HDAC-4, histone deacetylase 4; TIMP3, Tissue inhibitor of metalloproteinase 3; CPEB3, cytoplasmic polyadenylation element binding protein 3; SIRT1, sirtuin1; SOX9, SRY-box, transcription factor 9.

### 4.4 *In vivo*: treadmill training and running exercises

For a long time, the health promotion of the body through exercise has been widely recognized. Regular exercise can promote the metabolism of multiple tissues and organs in the human body and enhance the crosstalk between them, while also serving as a preventive and non-pharmacological strategy for many diseases (Qiu et al., 2023; Sato et al., 2022; MoTrPAC Study GroupLead AnalystsMoTrPAC Study Group, 2024). Under physiological conditions, the regulation of bones by motor stimuli no longer exists in a single form, but is a collection of different forms of force. These complex mechanical stimuli are essential for increasing bone density and maintaining its integrity, and activation of this event requires extensive metabolic and molecular remodeling including ncRNA.

In basic research, treadmill training has become a classic scheme for the construction of animal exercise models. 8-week exercise increased the mechanical strength of the femur and promoted the maturation of bone tissue in mice (Yang et al., 2021). The authors also analyzed the differentially expressed miRNAs identified in bone tissue with the differential expression profiles of miRNAs *in vitro* osteocytes. A total of eight miRNAs with similar expression trends may have played an important role in regulating bone metabolism (Yang et al., 2021). Similarly, with an 8-week moderate treadmill exercise intervention, rats exhibit higher osteogenic activity (upregulation of Runx2) and lower adipogenic activity (downregulation of PPARγ) (Qiu et al., 2021). It is important that the TGF-β pathway is activated, which is an important pathway that promotes osteogenic differentiation. In addition, five upregulated miRNAs and four downregulated lncRNAs were identified in the rat femur and tibia (Qiu et al., 2021). In conclusion, treadmill exercise-induced ncRNAs may be involved in bone metabolism in rodents.

*In vitro* loading experiments have demonstrated the sensitivity of miR-138-5p to mechanical stimuli. *In vivo*, 4 weeks of treadmill exercise increased the rate of bone formation in 2-month-old mice and inhibited miR-138-5p levels (Chen et al., 2020). In contrast, these phenomena were not significantly different in transgenic mice (TG) with miR-138, suggesting that high miR-138-5p levels in bone deprived TG mice of their response to mechanical load. Interestingly, the effect of the antagomir-miR-138-5p combined exercise intervention group was superior to that of the single intervention (Chen et al., 2020). Similarly, Yuan et al. (2019) found a downregulation of miR-214 levels in the tibia of young mice after 5 weeks of moderate-intensity exercise, as well as in primary osteoblasts treated with mechanical strain loading. On the contrary, the promotion of bone structure density and osteogenic factors, as well as the inhibition of osteoclasts, indicated the positive effect of exercise and loading on bone regulation. Overexpression of miR-214 severely inhibits the osteogenic process (Yuan et al., 2019). In addition, exercise can also ameliorate diabetes-related osteoporosis by inhibiting miR-150 to induce the expression of its target gene irisin (Behera et al., 2022). Therefore, these miRNAs may be targeted for the prevention of osteoporosis under the inhibition of exercise.

Similarly, lncRNAs also mediate exercise regulation of bone metabolism. To uncover the underlying mechanism of lncRNA in exercise in improving osteoporosis, Guo et al. (2022) performed genetic microarray analysis in mice that included ovarian resection and exercise intervention. The upregulation of lncRNA H19 in a large number of enriched lncRNAs is exciting. This is because the team has shown in previous studies that high-intensity exercise can trigger knee cartilage injury and reduce the expression of lncRNA H19 in cartilage, while 4 weeks of moderate-intensity treadmill rehabilitation can alleviate high-intensity exercise-induced traumatic osteoarthritis by reversing lncRNA H19 expression (Zhou X. et al., 2021). In addition, recent studies have shown that 9 weeks of moderate-intensity treadmill exercise attenuates bone loss due to oophorectomy by increasing the expression of lncRNA H19 and activating the Wnt/β-catenin pathway (Liu et al., 2024). Previously, the promotion of osteogenic differentiation by lncRNA H19 has been demonstrated by targeting multiple miRNAs, and the sensitivity of lncRNA H19 to mechanical loading and unloading *in vitro* and its response to exercise together underscore its prominent role in exercise alleviation of osteoporosis.

In order to study the effect of acute exercise on osteogenic differentiation after acute exercise, Valenti et al. (2019) collected the pre- and post-race serum of 20 male volunteers participating in the half marathon and added them to human mesenchymal stem cells. For selected miRNAs, the serum intervention promoted osteogenic differentiation-related miRNA expression (miR-21-5p, miR-129-5p, and miR-378-5p) while inhibiting adipogenic differentiation-related miRNAs (miR-188-5p). These miRNAs and target genes, especially miR-21-5p, promote osteogenic differentiation by targeting PTEN and SMAD7 to activate AKT and SMAD pathways (Valenti et al., 2019). In conclusion, appropriate exercise (running) alleviates bone loss by stimulating bone formation. Mechanistically, mechanosensitive miRNAs and lncRNAs may play a key role, which positively addresses the risk of osteoporosis.

### 4.5 *In vivo*: vibration and resistance movement

In recent years, whole-body vibration, especially at low amplitude and high frequency, has been shown to be a non-pharmacological method for the prevention and treatment of osteoporosis (Oliveira et al., 2016; de Oliveira et al., 2023). It is well known that bone strength and mechanical properties gradually decline with age. Yu et al. (2020) used high-throughput sequencing to identify differentially expressed miRNAs in BMSCs in young rats (6 months old) and old rats (20 months old). RT-qPCR verified that the levels of seven differential miRNAs were reduced in aged mice and that these reductions did not exhibit sexual dimorphism. Gain-of-function and loss-of-function experiments showed that miR-378a-3p had the most significant effect. Meanwhile, low-magnitude vibration (0.3g, 90 Hz) induced osteogenic differentiation of primary BMSCs in aged rats, thereby improving bone mineral density and osteogenic marker expression in age-related bone loss rat models (Yu et al., 2020). This frequency has been proven to be safe and effectively enhance osteogenic differentiation (Lau et al., 2010; Muir et al., 2013). In contrast, inhibition of miR-378a-3p reversed these phenomena, suggesting that low-amplitude and high-frequency vibrations induce osteogenic differentiation of BMSCs derived from older rats by upregulating miR-378a-3p. Mechanistically, miR-378a-3p targets and inhibits growth factor receptor-binding protein 2 (Grb2), which significantly increases the mRNA level of Ocn in response to siRNA (Yu et al., 2020). Consistent results were observed *in vivo* at the same vibration frequency as used *in vitro* (Yu et al., 2020). This suggests the role of vibration in the prevention and treatment of age-related bone loss.

When applied to the vibration intervention in human studies, whole-body vibration was found to be a sufficient mechanical condition to alter circulating miR-21-5p expression in postmenopausal women. However, in this study, none of the significant miRNAs *in vivo* were elicited at any time points after high-intensity acute resistance exercise (Buchanan et al., 2022). In contrast, previous studies have shown that in young men, miR-21-5p levels acutely decreased immediately after resistance exercise and then returned to baseline levels 60 min after exercise. However, this was limited to muscle tissue, and there were large differences among individual participants (Cui et al., 2017). In addition, with increasing age, circulating miRNA expression may respond differently to acute high-intensity resistance exercise, manifesting as age-related polarization (Margolis et al., 2017). Similar to acute resistance training, 12 weeks of long-term resistance training showed no significant difference in bone mineral density, markers of bone turnover, or serum miR-133 and miR-206 levels in women (aged 65–80 years) with sarcopenic obesity (Banitalebi et al., 2021). However, the given frequency of exercise (three times a week) may be the reason why resistance interventions are ineffective; after all, factor expression at the cyclic level quickly returns to baseline levels, as reported by Buchanan et al. (2022). Therefore, for exercise prescriptions in older adults or rehabilitation in older patients, the threshold for skeletal response to mechanical load should be considered based on age (Carrick-Ranson et al., 2022), rather than gender. This is because the 8-week exercise regimen failed to induce effective changes in several bone metabolism-related miRNAs and osteolipogenesis factors, either for strength or endurance training (Farsani et al., 2019). This is consistent with the results observed by Chen et al. (2020). This suggests that the bones are less sensitive to exercise interventions, possibly due to age-related physiological adaptations that lead to a decline in physical function.

**FIGURE 1:**
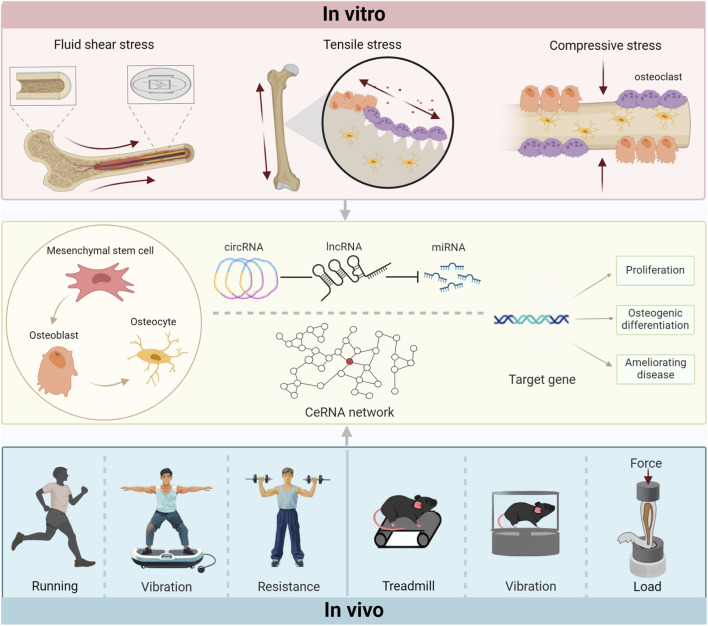
The force of contact regulates target genes by controlling the ceRNA network through various *in vitro*intervention modes and *in vivo*action mechanisms, subsequently affecting the bones.

In summary, exercise intervention has certain benefits in the regulation of bone and the improvement of homeostasis imbalance in bone metabolism, and ncRNAs are involved in the occurrence of these events. However, more potential mechanisms remain to be explored to elucidate the effects of active and passive movements on pathological conditions. In particular, age-related bone loss can be effectively alleviated by mechanical stimulation. In conclusion, these results verify the application value and prospect of exercise in the prevention and treatment of bone-related diseases (Figure 1).

## 5 NcRNA mediates mechanical unloading and regulates bone metabolism

### 5.1 Rodents: hindlimb unloading and simulated microgravity

In contrast to mechanical loading, unloading is a form of non-contact force. The unloading of bones can cause severe disuse osteoporosis, one of the main problems of bone health. This condition is common in astronauts on long-term space flights as well as in bedridden patients. Given the high cost of aerospace research and the mobility of bedridden patients, researchers have developed a variety of forms of microgravity simulation to explore the mechanical microenvironment. In animals, the hindlimb unloading (HU) model is a well-tolerated method and has been widely used to simulate the removal of bone bearing in space (Morey-Holton and Globus, 1998; Morey-Holton and Globus, 1985; Globus and Morey-Holton, 1985). Because microgravity not only promotes the catabolism of bones (Nabavi et al., 2011; Coulombe et al., 2020), but also impairs the adaptation of the cytoskeleton to mechanical stimuli. As an important factor in the load-bearing structure and mechanical transduction of cells, the cytoskeleton is particularly sensitive to external stimuli, especially changes in gravity (Qin et al., 2020; Klein-Nulend et al., 2012; Wu et al., 2022). This suggests that the inhibition of osteogenic differentiation in microgravity is partly caused by the loss of mechanical transduction ability, which may explain the occurrence of unload-induced osteoporosis and other diseases. *In vitro*, the use of clinostats, such as the Random Positioning Machine (RPM) or the Rotating Wall Vessel (RWV), simulates space weightlessness by continuously rotating around a horizontal axis to produce a vector weightlessness that prevents the cell from feeling gravity (Patel et al., 2007; Rauh et al., 2011). The sensitivity of osteoblasts and mesenchymal stem cells to mechanistic cues highlights the importance of clonal proliferation and osteogenic differentiation in preventing load-induced osteopenia and bone microstructural deterioration (Lee et al., 2017). As more and more ncRNAs are found to be involved in and regulate a variety of bone pathological processes, especially the role of miRNAs that maintain a well-conserved type among different species and the role of the ceRNA network mechanism formed by lncRNA and miRNA in the process of transcription or regulatory factors involved in osteogenic differentiation. Importantly, these ncRNAs are also involved in the transduction pathway of osteogenic differentiation and bone formation guided by mechanical stimulation. This underlines the stifling effect of mechanical loading on phenomena such as osteoporosis.

As discussed in the stretch section, miR-103a is a mechanosensitive miRNA whose expression is inhibited under tension and upregulated after 28 days of suspension (Zuo et al., 2015). Although no differences in body weight were exhibited, the bone tissue of the hanging mice was significantly thinner and the fracture vulnerability was increased (Zuo et al., 2015). Importantly, mechanical unloading-induced bone loss is partially counteracted by inhibitory treatment of miR-103a (Zuo et al., 2015). This may be due not only to the protein level of Runx2, the target gene of miR-103a, but also to the expression of Cav1.2, a subunit of L-type voltage-sensitive calcium channels (LTCC), which was increased by the inhibition of miR-103 in a simulated microgravity environment (Sun et al., 2015a; Sun et al., 2015b). The imbalance of the cell cycle may lead to abnormal osteoblast proliferation, but the mechanism in the unloading environment needs to be fully characterized. Sun Z. et al. (2019) found that cell cycle arrest in osteoblasts was associated with decreased expression of cyclin B1 when exposed to a tilt-rotating device for 48 h. This suggests that the mitotic index of osteoblasts is reduced in a simulated microgravity environment. In contrast, miR-181c-5p, which is also upregulated in microgravity, significantly inhibited the downregulation of cyclin B1 protein expression in microgravity-induced osteoblasts, thereby restoring impaired proliferation levels (Sun Z. et al., 2019).

The team also identified another miRNA, miR-132-3p. miR-132-3p was significantly upregulated and negatively correlated with osteogenic differentiation in rat bone tissue and weightless primary rat osteoblasts *in vitro* after 3 weeks of hindlimb unloading (Hu et al., 2015). This phenomenon has also been observed in mice and primary BMSCs that mimic weightlessness *in vitro* and *in vitro* (Hu et al., 2020). Notably, miR-132-3p appears to be upregulated under both loading (Liu M. et al., 2019) and unloading (Hu et al., 2015; Hu et al., 2020) conditions. Further studies confirmed that the E1A-binding protein p300 (Ep300), as a histone acetyltransferase important for Runx2 activity and stability, is a direct target of miR-132-3p (Hu et al., 2015). However, miR-132-3p can be upregulated by mimetic microgravity, in part, by decreasing Ep300 protein expression to inhibit osteoblast differentiation (Hu et al., 2015). Importantly, targeted inhibition of miR-132-3p effectively preserves bone mass, microstructure, and strength by promoting osteogenic differentiation in hindlimb unloading mice (Hu et al., 2020). Similarly, miR-494, miR-92b-3p, and miR-212-3p all mediate mechanical unloading-induced inhibition of osteogenic differentiation through different mechanisms, providing more potential protective strategies for disuse osteoporosis (Qin et al., 2019; Zhang et al., 2022; Zhang et al., 2023). miR-208a-3p inhibited the differentiation of osteoblast MC3T3-E1 by targeting ACVR1. In turn, therapeutic inhibition of miR-208a-3p *in vitro* rescues unload-induced bone loss by increasing bone formation and trabecular bone microstructure (Arfat et al., 2018). However, unlike miRNA-132-3p (Hu et al., 2020), miR-208a-3p antagonist tail vein injections were received not only for three consecutive days prior to suspension, but also during the last week of unloading to maintain high concentrations of the antagonist (Arfat et al., 2018). This suggests that the targeted silencing miRNA strategy has great potential in the prevention and treatment of unloading-induced disuse osteoporosis, and may become a potential target for clinical application.

In an interesting experiment, 8-week-old mice were unloaded for 4 weeks before returning to the ground for 2 weeks (Chen et al., 2020). With the extension of unloading time, the level of miR-138-5p level increased significantly. In contrast, bone mineral density, bone mass, and osteogenic marker genes decrease. These changes were partially restored during the reloading period in mice (Chen et al., 2020). *In vitro*, the reduction of the osteogenic marker genes Alp and Col1a1 in primary osteoblasts under microgravity was also restored by miR-138-5p antagonist treatment, which was achieved by targeting microtubule-actin cross-linking factor 1 (MACF1) (Chen et al., 2020). MACF1 acts as a cytoskeletal cross-linking agent and promotes the proliferation and differentiation of osteoblasts (Yin et al., 2018; Hu et al., 2018; Qiu et al., 2020). In addition, silencing miR-138-5p partially attenuates microgravity-induced inhibition of osteoblast proliferation and promotion of apoptosis by targeting SIRT1 (Xu L. et al., 2023). Although there are still many uncertainties about the role of miR-138-5p in regulating the breakdown and anabolism of chondrocytes (Brito et al., 2023), it is clear that it at least inhibits the differentiation of osteoblasts. As such, targeted osteoblast delivery of miR-33-5p (Wang H. et al., 2018) and miR-133a (Zhou Y. et al., 2021) contribute to the anti-osteopenic effect, thereby alleviating the challenge of mouse bones to the HU model. Therefore, treatment with inhibitors of mechanical load-sensitive miRNAs may be an effective strategy for restoring bone mass in bedridden patients who are unable to effectively undergo mechanical stimulation. For people who need rehabilitation training after surgery, miRNA bone targeting inhibition and mechanical stimulation may be an effective means to rapidly promote bone formation.

Although the role of many lncRNAs in osteogenesis has been widely reported, only a few studies have shown that lncRNAs are involved in the occurrence and progression of unloading-induced bone loss (Li B. et al., 2017). With Hu et al. (2017) the genome-wide prediction and analysis of lncRNAs under microgravity conditions was carried out for osteoblast differentiation. There is a growing understanding of lncRNAs and their competitive binding to miRNAs to regulate unloading-induced bone loss. lncRNA ODSM is an osteoblast differentiation-associated lncRNA whose expression was significantly reduced in the femur of mice suspended for 21 days (Wang Y. et al., 2020). *In vitro* studies have shown that overexpression of lncRNA OSDM mitigates apoptosis induced by simulated microgravity and promotes the mineralization of MC3T3-E1 (Wang Y. et al., 2020). This may be partially dependent on the targeted regulation of ELK1 by miR-139-3p (Wang et al., 2018c). At the same time, targeted overexpression of lncRNA OSDM also partially reversed the bone loss exhibited by mice after mechanical unloading (Wang Y. et al., 2020). Similarly, lncRNA OGRU was significantly reduced in MC3T3-E1 cells under hindlimb unloading mouse bone specimens and oblique spin unloading conditions (Wang K. et al., 2020). As a ceRNA, lncRNA OGRU promotes protein expression of the latter through the miR-320-3p/Hoxa10 axis, thereby promoting osteoblast activity and matrix mineralization under *in vitro* and *in vivo* loading conditions (Wang K. et al., 2020). ELK1 and Hoxa10 have been shown to regulate various cellular processes and are closely related to osteogenic differentiation (Zhang et al., 2009; Hassan et al., 2007). In addition, EKL1 can also induce the antisense lncRNA of Hoxa10 to ameliorate lung adenocarcinoma progression (Sheng et al., 2018). Considering that the encapsulation of the tail of the mouse by the unloading experiment led to the attenuation of the drug delivery effect, the targeted delivery of lncRNA ODSM and lncRNA OGRU was continuously injected in the tail vein 3 days before the suspension implementation. To better deliver lncRNAs to osteoblasts, the researchers used the (DSS)_6_-liposome delivery system, which has a high affinity for low-crystalline hydroxyapatite and calcium phosphate compounds on the bone-forming surface, enabling targeted drug delivery to osteoblasts or mesenchymal stem cells without significant cytotoxicity (Yarbrough et al., 2010; Gao et al., 2022).

A recent report suggests that lncRNA MALAT1 is downregulated in hindlimb unloading (HU) mice and MC3T3-E1 cells treated with simulated microgravity (MG). In MG cells, lncRNA MALAT1 promotes osteogenic differentiation and inhibits apoptosis (Zhou et al., 2022). The luciferase reporter gene showed that miR-485-5p was identified as a target for lncRNA MALAT1, while WNT7B was identified as a target for miR-485-5p. Overexpression of lncRNA MALAT1 alleviates bone loss in vitro and *in vivo* models (Zhou et al., 2022). This is consistent with the effect shown in a previous report (Gao et al., 2018). However, the injection of the drug was still administered before the model was established, but the injection site was no longer the tail vein; instead, it was directly and vertically injected into the lateral thigh muscles of the mice. In addition to osteogenic differentiation of osteoblasts, osteogenic differentiation of bone marrow mesenchymal stem cells is also essential to prevent the development of osteoporosis. Extraction of primary BMSCs from unloaded mice reveals that lncRNA HCG18 expression is upregulated. Lentivirus-mediated shRNA transfection was used to knock down lncRNA HCG18 and inject it into vivo, which alleviated hindlimb unloading (HU)-induced bone loss (Che et al., 2020). This inhibits the osteogenic differentiation of HU-induced BMSCs by the miR-30a-5p/Notch1 axis. However, the authors do not indicate how and when the drug is injected (Che et al., 2020). The above evidence provides the mechanosensitivity of a variety of lncRNAs, with altered levels *in vitro* and *in vivo* under loading conditions. Targeted delivery of lncRNAs may effectively alleviate hindlimb suspension-induced bone loss, highlighting that lncRNAs may be a promising therapeutic target for alleviating osteoporosis. However, to date, little is known about the osteogenic differentiation of circRNAs in microgravity. Several circRNAs that have been identified remain unwell characterized (Cao et al., 2021; Table 4).

**TABLE 4:** Hindlimb unloading/Simulated microgravity regulates the effects of ncRNAs on bone tissue or cells.

Species	Cell type	Mechanical loading conditions	ncRNAs	Target gene	Phenotype
Mouse	Bone tissue (Zuo et al., 2015)	HU, 28d	miR-103a↑	RUNX2	bone loss
Mouse	OBs (Sun et al., 2019b)	MG, 24rpm, 48 h	miR-181c-5p↑	cyclin B1	osteoblasts proliferation↓
Rat	Bone tissue prOBs (Hu et al., 2015)	HU, 21d MG, 30rpm	miR-132-3p↑	Ep300	osteogenic differentiation↓
Mouse	Bone tissue BMSCs (Hu et al., 2020)	HU, 21d MG, 24rpm	miR-132-3p↑	n.s	osteogenic differentiation↓
Mouse	C2C12 OBs (Qin et al., 2019)	HU, 28d MG, 72 h	miR-494↑	BMPR2 RUNX2	osteogenic differentiation↓
Mouse	MC3T3-E1 (Zhang et al., 2022)	MG, 48h, 24rpm	miR-92b-3p↑	ELK4	osteogenic differentiation↓
Mouse	MC3T3-E1 hFOB (Zhang et al., 2023)	MG, 24rpm, 24/48/72 h	miR-212-3p↑	Hmgb1	osteogenic differentiation↓
Mouse	Bone tissue MC3T3-E1 (Arfat et al., 2018)	HU, 28d MG, 8rpm, 48 h	miR-208a-3p↑	ACVR1	osteogenic differentiation↓
Mouse	Bone tissue OBs (Chen et al., 2020)	HU, 28d MG, 8rpm, 24/48 h	miR-138-5p↑	MACF1	bone loss
Mouse	MC3T3-E1 (Xu et al., 2023a)	MG, 48h, 24rpm	miR-138-5p↑	SIRT1	osteoblast proliferation↓ osteoblast apoptosis↑
Mouse	Bone tissue (Wang et al., 2018b)	HU, 21d	miR-33-5p↑	n.s	osteogenic differentiation↓
Mouse	Bone tissue (Zhou et al., 2021c)	HU, 21d	miR-133a↑	n.s	osteogenic differentiation↓
Rat	Bone tissue (Li et al., 2017b)	HU, 28d	lncRNA-H19↓	Dkk4	Wnt signaling↓ osteogenic function↓
Mouse	MC3T3-E1 (Hu et al., 2017)	MG, 24rpm 24/48/72 h	857 lncRNAs 2,264 mRNAs	n.s	osteogenic differentiation↓
Mouse	Bone tissue MC3T3-E1 (Wang et al., 2020a, Wang et al., 2018a)	HU, 21d MG, 24rpm, 48 h	lncRNA ODSM↓ miR-139-3p↑	ELK1	osteoblast apoptosis↑ osteogenic differentiation↓
Mouse	Bone tissue MC3T3-E1 (Wang et al., 2020b)	HU, 21d MG, 24rpm, 24/48/72 h	lncRNA OGRU↓ miR-320-3p↑	Hoxa10	bone loss
Mouse	Bone tissue MC3T3-E1 (Zhou et al., 2022)	HU, 21d MG, 28rpm, 15min	lncRNA MALAT1↓ miR-485-5p↑	WNT7B	osteogenic differentiation↓ apoptosis↑
Mouse	Bone tissue (Che et al., 2020)	HU, 21d	lncRNA HCG18↑ miR-30a-5p↓	NOTCH1	osteogenic differentiation↓

HU, hindlimb unloading; MG, microgravity; RUNX2, runt related transcription factor 2; Ep300, E1A binding protein p300; BMPR2, bone morphogenetic protein receptor type 2; ELK4, ETS; transcription factor ELK4; Hmgb1, High mobility group box 1; ACVR1, activin A receptor type 1; MACF1, microtubule actin crosslinking factor 1; SIRT1, sirtuin1; Dkk4, dickkopf WNT; signaling pathway inhibitor 4; ELK1, ETS; transcription factor ELK1; Hoxa10, homeobox A10; WNT7B, wingless-type MMTV; integration site family; member 7B; NOTCH1, notch receptor 1.

### 5.2 Humans: bed rest

Mechanical stimulation is crucial for bones to maintain their density and quality. On the contrary, long-term mechanical unloading means the disappearance or significant reduction of mechanical stimulation signals. When strictly resting in bed, the weekly bone loss rate can reach 1%–2%. In addition, the bones have become more fragile. In a clinical human study, Ling et al. (2017) performed −6° head-down tilt bed rest on 16 young male volunteers (aged 20–32 years) for 45 days. Bedrest individuals had significantly lower levels of 11 circulating miRNAs compared to baseline. The levels of three miRNAs (miR-103, 130a and 1,234) in the plasma of volunteers were significantly increased after 10 days of recovery. Interestingly, miR-151-3p levels continue to decline. However, only miR-1234 was significantly correlated with most of the skeletal parameters of bedridden individuals (Ling et al., 2017). This suggests that circulating miR-1234 may be a potential biomarker of bed rest-induced bone loss. Another bedridden experiment with 11 adults (6 males, five females) aged 25–50 years found that four targeted miRNAs were significantly correlated with biochemical markers of bone metabolism at baseline (Bemben et al., 2021). Although there are sex differences in the percentage of bone mineral density and several bone biomarkers in the hip joint after bed rest, this phenomenon has not been observed in miRNAs, which is consistent with previous conclusions (Kelch et al., 2017). miR-21-5p was the only miRNA that was significantly upregulated after bed rest, and its magnitude was positively correlated with serum calcium content (Bemben et al., 2021). However, the upregulation (Kelch et al., 2017; Seeliger et al., 2014; Mohammadisima et al., 2023) or downregulation (Yavropoulou et al., 2017; Zhao et al., 2019) of miR-21 expression in osteoporosis patients in different samples has undoubtedly increased the efforts to further elucidate the biological significance of osteoporosis and its complex mechanisms. Although the elevation of miR-21-5p was equally observed in mice after unloading by Chen et al. (2020) they seem to pay more attention to the highest elevated miR-138-5p. In the long-term bed rest survey, it was found that the expression of miR-138-5p increased with the prolongation of bed rest time in the bone specimens of bedridden men (50–59 years old). In contrast, RT-qPCR showed that osteogenic marker gene expression (Alp and Bglap) and BMD (T-score) were inversely correlated with ambulation (Chen et al., 2020). In conclusion, the above evidence suggests that the mechanosensitivity of certain miRNAs in human samples reflects their metabolic response to bone formation, and targeting these miRNAs may be an effective strategy to ameliorate disuse osteoporosis.

**FIGURE 2:**
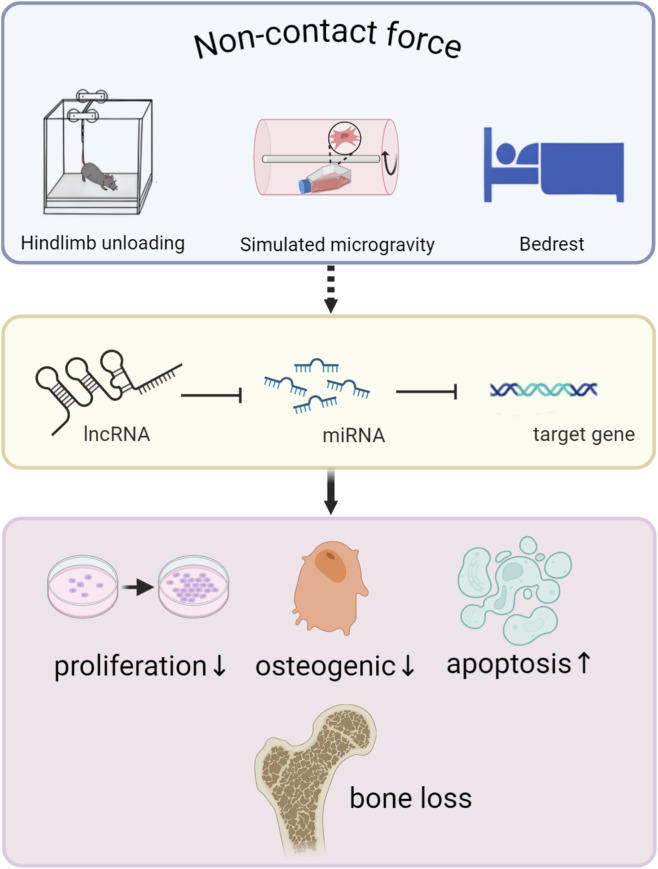
The non-contact force component alleviates the bone’s sensitivity to force via ncRNAs, ultimately resulting in bone loss.

Overall, improving bone loss caused by mechanical offloading is still a major clinical challenge, and the value of mechanosensitive ncRNAs as new targets in the treatment of OP has attracted increasing attention. With the help of a variety of developed instruments, microgravity simulation at the cellular level is being explored. In addition, a new large-gradient superconducting magnet that can be used at the cellular level may be an effective terrestrial gravity simulator (Qian et al., 2012; Sun YL. et al., 2015). However, there is relatively little information about it. In the future, it is still necessary to continue to reveal the form of mechanical loading, as well as the comprehensive impact of load intensity and frequency on bones, and develop ncRNA delivery vectors to improve bioavailability and clarify the residence time of drugs *in vivo*. After all, it is difficult for people to predict the occurrence of diseases and realize the importance of preventing them. Although ncRNAs have achieved promising results in translational medicine related to bone tissue engineering (Zhang et al., 2012; Guan et al., 2022; Meng et al., 2023; Xu H. et al., 2023), there is a need to further explore the molecular mechanisms. It is believed that in the near future, as the mechanism and network of ncRNA are revealed one after another, it will make it a more promising OP therapeutic drug. In conclusion, understanding the mechanism of bone damage caused by waste and load and the underlying mechanisms behind it is an important area of bone biology, as well as providing a reliable therapeutic strategy for finding solutions to osteopathology (Figure 2).

## 6 Discussion

Although this study systematically reviewed the significant role of ncRNAs in the regulation of bone metabolism by mechanical load and revealed various mechanically sensitive ncRNAs and their underlying mechanisms, there are still several limitations in this field that are worthy of in-depth exploration. Firstly, ncRNAs exhibit significant versatility and complexity in mechanical conduction. Different types of ncRNAs, and even different members of the same category, often show differences or even conflicts in their expression changes and functions when responding to various forms of mechanical stimuli such as fluid shear stress, tension, and compression. For instance, miR-132-3p was upregulated in both mechanical stretching and simulated microgravity unloading models, but they respectively led to two seemingly contradictory phenotypes: inhibition and promotion of osteogenic differentiation. This indicates that the mechanical biological functions of ncRNA are highly dependent on the specific cellular microenvironment and mechanical background, and the potential regulatory network behind it may be far more complex than currently known. Secondly, in the current research, the standardization issue of mechanical stimulation parameters is particularly prominent. Due to the significant differences in mechanical loading modes, intensities, frequencies and action durations adopted by various studies, the comparability among different research results is extremely low. Even in the same loading mode, the parameter standards may differ by tens of times. For instance, the tensile strain parameters that promote osteogenic differentiation range from 2000 µε to 80,000 µε in the literature. This inconsistency of parameters makes it difficult to determine the thresholds of truly “physiological” and “non-physiological” mechanical stimuli, and also hinders the establishment of reliable *in vitro* mechanical models to accurately simulate *in vivo* processes. Finally, there are inherent differences in the sensitivity of different cell types to mechanical stimuli, which is determined by their own physical locations. Therefore, the response mechanisms to the same mechanical stimulus and the subsequent biological effects are also different. For example, the expression of miR-29b-3p in osteocytes was downregulated after mechanical stretching, but it did not respond to osteoblasts after stretching. Furthermore, most studies have only focused on a certain type of cell and lack systematic research on the mechanical signal dialogue between different cells, which limits our understanding of the overall network mechanism by which mechanical load regulates bone metabolism.

## 7 Summary and prospect

The potential connection between bones and mechanical forces has long been explored by researchers. A major goal of skeletal biomechanics is to integrate the physiological and biochemical responses produced by osteocytes in response to mechanical stimuli and adapt to the properties of their coordination mechanisms. While the consensus that mechanical stimulation is beneficial to bone growth is widely recognized, more and more *in vitro* models are being promoted and applied. Although these *in vitro* mechanical simulations cannot realistically reproduce the complex physiological regulatory networks *in vivo*, they greatly enhance our understanding of the mechanical transduction of bone cells.

Motion-induced mechanical stimuli are complex for the human organism, but independent studies collectively show that skeletal remodeling events occur in response to mechanical stimuli. Although this must take into account age, it is important that in the future there is a need to quantitatively assess the magnitude of the load *in vitro* and to identify the magnitude difference between the *in vitro* and extracorporeal load, so that the force closest to the physiological state can be applied *in vitro* stimulation to elucidate the underlying mechanism of mechanical loading. These findings will provide new insights into bone mechanotransduction and also open up the possibility of using ncRNAs to modulate bone tissue engineering in translational medicine.

A variety of miRNAs and lncRNAs have been identified to modulate osteogenic hallmark genes, enhance bone microstructure and mechanical strength to promote bone formation (Lian et al., 2012; Ju et al., 2019; Aurilia et al., 2021; Wang et al., 2019), and alleviate bone disease by targeting multiple pathways (Ko et al., 2020; Xue et al., 2023; Zhao et al., 2024). This reflects the important role of ncRNAs in regulating bone metabolic homeostasis. According to current reports, not only can a single lncRNA target multiple miRNAs, but a miRNA can also have multiple downstream target gene binding sites. In addition, the functional mechanisms of circRNA in osteogenic differentiation have been described, and their involvement in a variety of physiological and pathological states has been revealed (Huang et al., 2022; Mazziotta et al., 2024). However, research on circRNAs in response to different forms of mechanical loading remains in its infancy. Their closed-loop structure confers higher stability, making them promising ideal mechanosensitive biomarkers or therapeutic targets. At present, a large number of mechanosensitive ncRNAs have been enriched in bone tissue by high-throughput sequencing, but their functional roles have not been well characterized in the mechanical environment, and future studies need to further explore and elucidate the mechanism of mechanosensitive ncRNAs in bone metabolism. The revelation of these ncRNAs contributes to a better understanding of how bones transmit mechanical signals to meet the need to adapt to the mechanical environment.

Currently, it is still urgently necessary to systematically clarify the core regulatory role of ncRNA networks in mechanical transduction to accelerate its clinical transformation process. In the future, a more comprehensive mechanical biological regulatory map should be constructed relying on multi-omics technologies. However, although ncRNAs treatment strategies have shown significant potential in animal models, their clinical translation still faces severe challenges such as delivery efficiency, tissue targeting, and long-term safety. Therefore, continuing to focus on developing efficient bone-targeted delivery systems will be an important research direction for enhancing the accuracy and feasibility of treatment.

Finally, the important role of mechanical loading in regulating bone adaptation and determining the structural integrity of bones is widely recognized. In this review, we have discussed the effects of multiple forms of mechanical stimulation, including contact and non-contact forces, on bone *in vitro* and *in vivo*, and highlight the mechanisms of action of ncRNAs in these processes. In conclusion, a large number of ncRNAs have been shown to be mechanosensitive, but we have only discussed their performance in the skeletal mechanistic environment. With the deepening of research, it is believed that more ncRNAs with mechanical sensitivity will be revealed in the future, which will provide a theoretical basis for the application of ncRNA-mediated mechanical load in the field of bone biology, and also provide a potential therapeutic strategy for solving clinical bone diseases.
